# Abnormal maternal behavior in mice lacking phospholipase Cβ1

**DOI:** 10.1080/19768354.2022.2141319

**Published:** 2022-11-07

**Authors:** Hea-jin Kim, Jaewon Jang, Hae-Young Koh

**Affiliations:** a Brain Science Institute (BSI), Korea Institute of Science and Technology (KIST), Seoul, Republic of Korea; b Division of Bio-Medical Science & Technology, KIST School, Korea University of Science and Technology (UST), Seoul, Republic of Korea; c Department of Life Sciences, Korea University, Seoul, Republic of Korea

**Keywords:** Maternal neglect, maternal motivation, maintenance of maternal behavior, Phospholipase Cβ1, mice

## Abstract

Motherhood goes through preparation, onset and maintenance phases until the natural weaning. A variety of changes in hormonal/neurohormonal systems and brain circuits are involved in the maternal behavior. Hormones, neuropeptides, and neurotransmitters involved in maternal behavior act via G-protein-coupled receptors, many of which in turn activate plasma membrane enzymes including phospholipase C (PLC) β isoforms. In this study, we examined the effect of PLCβ1 knockout (KO) on maternal behavior. There was little difference between PLCβ1-KO and wild-type (WT) dams in the relative time spent in maternal behavior during the period between 24 h prepartum and 12 h postpartum (−24 h ∼ PPH 12). After PPH 18, however, PLCβ1-KO dams neglected their pups so that they all died in 2–3 days. In the pup retrieval test, latency was not different during the period within PPH 12, but after PPH 18, PLCβ1-KO dams could not finish pup retrieval in a given time. During both periods, FosB expression in the nucleus accumbens (NAcc) of PLCβ1-KO dams was significantly lower than WT, but not different in the medial preoptic area (mPOA). Given that mPOA activity is required for initiation of maternal behavior, and that NAcc is known to be involved in maternal motivation and maintenance of maternal behavior, our results suggest that PLCβ1 signaling is essential for transition from the onset to maintenance phase of maternal behavior.

## Introduction

Maternal behavior follows preparation, onset, and maintenance before natural weaning occurs (Brunton and Russell 2008; Kristal 2009; Numan 2012). During the preparation, pregnant dams build a ‘brood’ nest and spend more time self-grooming than virgins. Upon parturition and exposure to the neonate stimuli, the immediate onset of maternal caretaking behavior is triggered by neurohormonal activation of the medial preoptic area (mPOA) that has been primed by dynamic hormonal milieu of the pregnancy and pregnancy termination (Numan 2007). Continuation or long-term enhancement in maternal behavior comes even under the changing stimuli and the reduced hormone levels later in the postpartum period. This maintenance is believed to be driven by maternal motivation (Lee et al. 2000; Wansaw et al. 2008) which is established after prolonged interaction with newborn pups. The mesolimbic dopamine (DA) system from the ventral tegmental area (VTA) to nucleus accumbens (NAcc) is critical in maternal motivation (Numan 2006; Numan and Stolzenberg 2009).

Neurobiological research on maternal behavior over the past half century has often focused on the onset but rarely on maintenance or ‘transition’ from the onset to maintenance. In a NAcc shell lesion study using rat, the lesion during pregnancy resulted in a normal onset but disturbed the maintenance (i.e. ‘maternal memory’), and the lesion 24 h after pup experience postpartum did not interfere with the maintenance. These results suggest that NAcc shell activation in 24 h postpartum is essential for consolidation of maternal responsiveness, but that an intact NAcc shell is not necessary for either the onset or the performance of maternal behavior after consolidation (Lee et al. 1999; Li and Fleming 2003). A model based on these findings proposes that NAcc shell activity during the pup experience within about 24 h postpartum may lead to neural modification in regions outside the NAcc (e.g. ventral pallidum) (Numan and Stolzenberg 2009). Failure to proceed to the maintenance phase, most likely by loss of maternal motivation, may result in maternal neglect (Strathearn 2011). Most gene-targetted mouse models display impaired/reduced performance of some elements of maternal behavior, but downright neglect after normal onset is rarely observed (Kuroda et al. 2011). There has been a study on a naturally occurring maternal neglect in the MaD1 mice, but it lacks detailed analysis of the peripartum behavior (Gammie et al. 2008), say, on whether or not onset does occur at all or when they begin neglecting pups.

Hormones, neuropeptides, and neurotransmitters involved in maternal behavior act via G-protein-coupled receptors, many of which in turn activate plasma membrane enzymes including phospholipase C (PLC) β isoforms (Sladek and Song 2012; Boumansour et al. 2021; Jang et al. 2021). The forebrain Gαq/11-deficient mice (Wettschureck et al. 2006) are known to neglect pups from the very start after parturition. PLCβ1, one of the downstream enzymes of Gαq/11, is expressed in the forebrain areas (Watanabe et al. 1998; Fukaya et al. 2008; Hozumi et al. 2008; Montaña et al. 2012) that overlap with some of those related to aspects of maternal behavior including maternal motivation (e.g. NAcc, medial prefrontal cortex, cingulate cortex, olfactory bulb, amygdala, lateral septum; perisynaptic sites of medium spiny neurons in NAcc, pyramidal cells in cortex and hippocampus, granule cells and mossy cells in dentate gyrus). The present study examined the effect of PLCβ1 knockout (KO) on maternal behavior. We found that PLCβ1-KO dams display normal preparation and onset, but neglect their young at about 18 h postpartum, suggesting that PLCβ1 signaling is not required for normal maternal behavior during the preparation and onset phases, but is necessary for proceeding to the maintenance phase. PLCβ1-KO mice can be a good mouse model for studying the process of transition from the onset to maintenance phase of maternal behavior.

## Materials and methods

### Subjects

Phospholipase Cβ1 (PLCβ1) wild-type (WT), knockout (KO) and heterozygous (HT) mice were obtained by crossing C57BL/6J (N30) PLCβ1^+/-^ and 129S4/SvJae (N40) PLCβ1^+/-^ mice as described previously (Kim et al. 1997; Kim and Koh 2016). Subjects were female mice 15–20 weeks old at the start of the experiment. Subjects were maintained on a 12:12 – light/dark schedule with light on at 08:30 AM. Animal care and handling followed institutional guidelines of Korea Institute of Science and Technology (KIST). All experimental protocols were approved by the Institutional Animal Care and Use Committee (IACUC) of KIST (AP201149).

### Maternal behavior protocol

Virgin females 15–20 weeks old (WT, *N* = 7; KO, *N* = 6) were mated with WT virgin males 20–30 weeks old. Each of the females was housed with its partner until it became visibly pregnant (around day 14 of pregnancy) and then was singly housed for video monitoring of peripartum behaviors. Behavioral analysis was performed on the basis of previously employed ethological parameters: ‘nesting’, ‘(self-) grooming’, ‘licking’, and ‘hovering’, as manifestations of maternal behavior (Lonstein and Fleming 2001). ‘Relative time spent in total maternal behavior’ was obtained as [time spent in nesting + grooming + licking + hovering] / [total recording time], every six hours during the 48-h video-recording period (24 h prepartum ∼ 24 h postpartum). Numbers of mice (*N*) are: (WT: *N* = 7 for all groups), (KO: −24 ∼ 0, *N* = 3; +6, *N* = 4; +12, *N* = 4; +18, *N* = 5; +24, *N* = 5). ‘Relative time spent in pup-directed maternal behavior’ was obtained as [time spent in licking + hovering] / [total recording time], every six hours for 24 h postpartum. Numbers of mice (*N*) are: (WT: *N* = 7 for all groups), (KO: 0, *N* = 6; +6, *N* = 4; +12, *N* = 4; +18, *N* = 5; +24, *N* = 5).

### Nest construction

Each of 20 WT and 13 KO dams was placed alone in the home cage with 3 × 3 cm pieces of cotton nesting material evenly spread on top of the ordinary wooden chips. An hour later, photographs were taken of the floor of each cage to inspect whether there was a nest made of the cotton material.

### Pup retrieval test (PRT)

20 WT, 13 KO, and 8 PLCβ1^+/-^ (HT) dams were tested repeatedly during the period within PPH 12 and after PPH 18. A dam was left alone with nesting material in the home cage for 1 h to build a nest first, and then three of its pups were put into three corners of the cage away from the nest. The dam was given 600 s to finish retrieving all three pus to the nest. The time taken for the dam to pick and carry each pup to the nest (latency) was measured. Numbers of mice (*N*) used are: PPH12 WT, *N* = 9; PPH18 WT, *N* = 11; PPH12 KO, *N* = 6; PPH18 KO, *N* = 8.

### Histology for FosB expression in the medial preoptic area, nucleus accumbens, and medial prefrontal cortex

The basic histochemistry procedure was the same as described in our previous publication (Kim and Koh 2021) which was modified from other literature (Perrotti et al. 2004; Zhang et al. 2006; Nunez et al. 2010). After perfusion with 10% formalin solution neutral-buffered, brains were removed and post-fixed in the same fixative for 24 h. Brain tissues were infiltrated with 30% sucrose overnight, frozen, and then sectioned into 40-μm sections, and the consecutive sections were placed in six-well plates containing PBS. Sections were floated in 3% H_2_O_2_ in methanol for 10 min, and then in PBS with 0.3% Triton X-100. After being in blocking serum in PBS for 1 h, sections were incubated with the polyclonal antibody to FosB protein (Santa Cruz Biotechnology; diluted 1:1000) in blocking serum at 4 °C for 3 days. After being washed in PBS, the sections were incubated in the biotinylated secondary antibody, anti-rabbit IgG (VECTASTAIN® Elite ABC kit) for 1 h, followed by incubation in an avidin and biotinylated horseradish peroxidase macromolecular complex (VECTASTAIN® Elite ABC kit) for 1 h. The sections were then rinsed in PBS, mounted on subbed slides, and cover-slipped with mounting medium (VECTOR, VectaMount). Medial preoptic area (mPOA), nucleus accumbens (NAcc), and medial prefrontal cortex (mPFC) were collected by a systematic and random sample of sections according to a brain atlas. Every 6–8th section from bregma 1.98∼1.42 mm for the mPFC, 1.54∼0.74 mm for the NAcc, and 0.14∼−0.1 mm for the mPOA, gave us approximately three random sections for each area per animal. Each area was photographed by using a microscope equipped with a digital camera (Olympus, Tokyo, Japan). The numbers of FosB-positive cells were counted within each brain area by using the Image-J free software (NIH, MD, United States). Bilateral counts per square mm from each section served as a data point. Sections that were damaged or difficult to count FosB-positive cells were excluded. Numbers of mice (*N*) and sections (*n*) used are: (Within PPH 12: mPFC WT, *N* = 3, *n* = 9; mPFC KO, *N* = 3, *n* = 8; NAcc WT, *N* = 4, *n* = 12; NAcc KO, *N* = 3, *n* = 7; mPOA WT, *N* = 4, *n* = 8; mPOA KO, *N* = 3, *n* = 3), (After PPH 18: mPFC WT, *N* = 14, *n* = 33; mPFC KO, *N* = 15, *n* = 41; NAcc WT, *N* = 14, *n* = 42; NAcc KO, *N* = 10, *n* = 28; mPOA WT, *N* = 9, *n* = 17; mPOA KO, *N* = 10, *n* = 24).

### Statistical analysis

**Figure 1.**
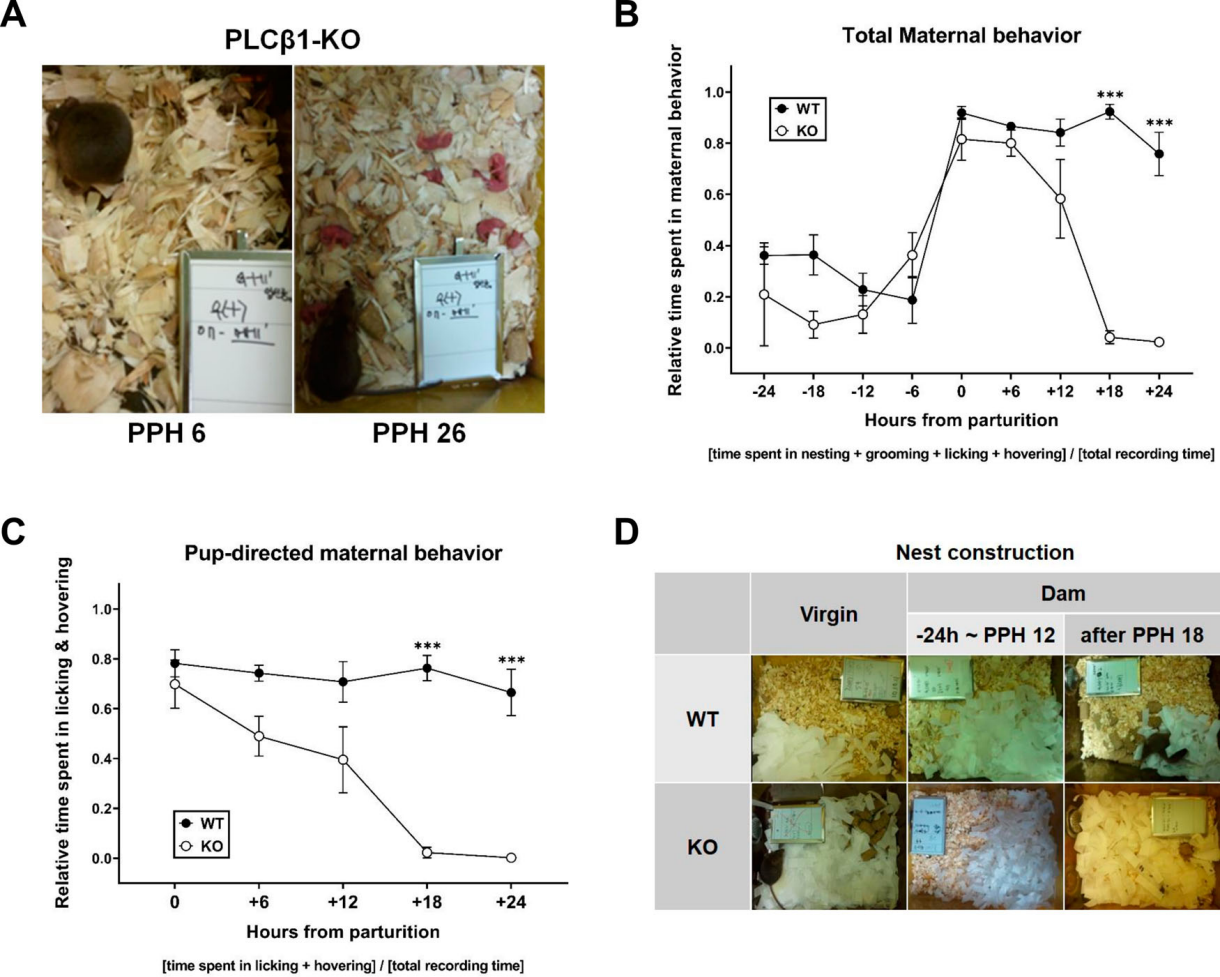
PLCβ1-KO dams neglected pups after postpartum hour 18 (PPH 18). (A) KO dam at PPH 6 (left) hovering over and nursing the pups, and at PPH 26 (right) neglecting pups that are left scattered all over the floor. (B) Total maternal behavior of WT (black circle) and KO (white circle) dams measured every six hours during the 48-h peripartum period. (C) Pup-directed maternal behavior of WT (black circle) and KO (white circle) dams measured every six hours during the 24-h postpartum period. (D) Representative photographs of nest construction. Top: by WT virgin females, WT dams during the period between 24 h prepartum and PPH 12 (−24 h ∼ PPH 12) or after PPH 18; Bottom center: by KO dams during −24 h ∼ PPH 12. All values are Mean ± SEM. ***p < 0.0001.

**Figure 2.**
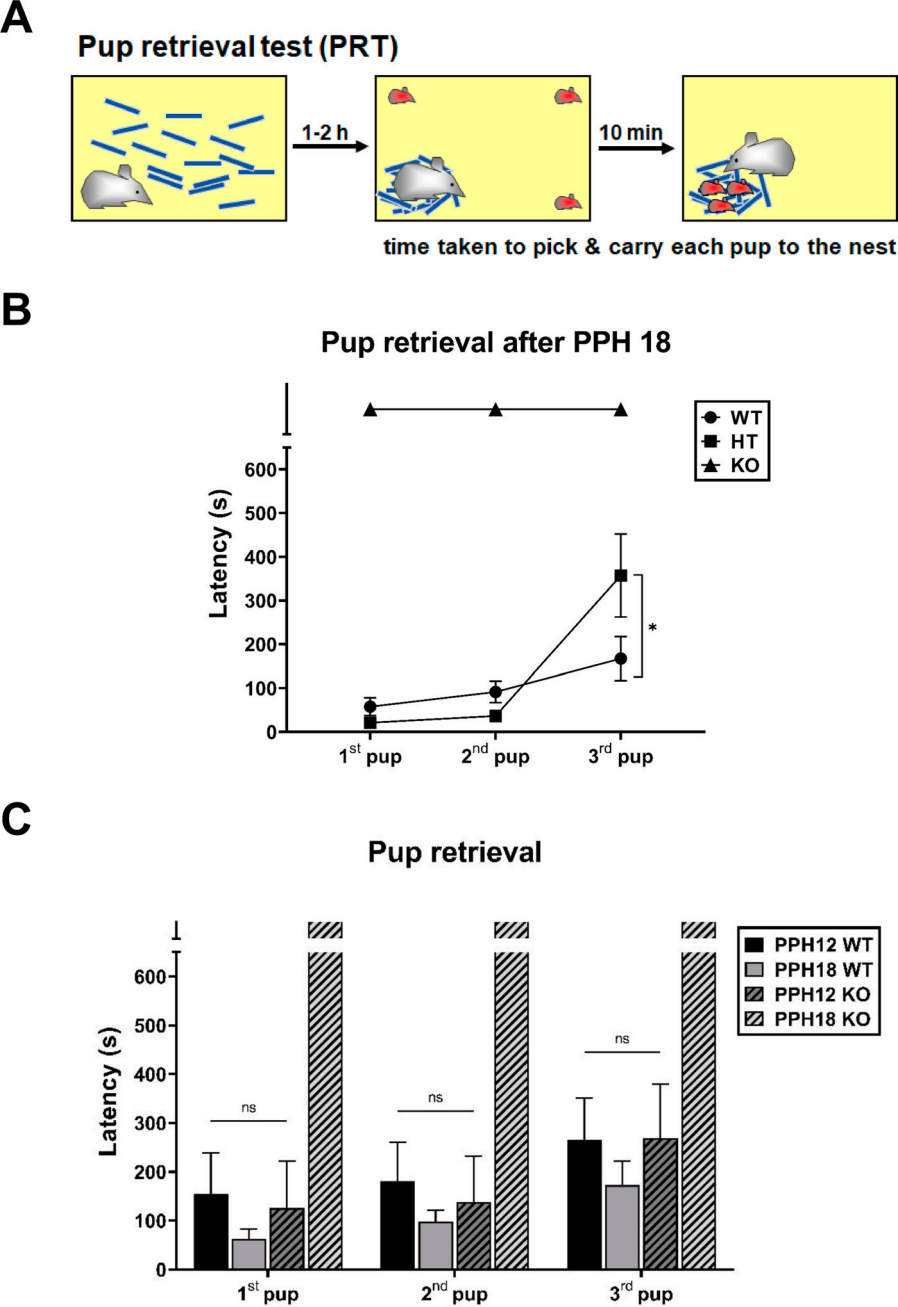
PLCβ1-KO dams could not finish pup retrieval task after PPH 18. (A) Pup retrieval test (PRT) method. (B) Pup retrieval by wild-type (WT, circle), heterozygous (HT, square) and KO (triangle) dams after PPH 18. (C) Pup retrieval by WT (plain) and KO (hatched) dams during the period within PPH 12 (PPH12, dark) and after PPH 18 (PPH18, light). All values are Mean ± SEM. *p < 0.05; ns indicates ‘not significant’.

Data were acquired using GraphPad Prism 7.03 software. Data for Figure 1(B,C) and 2(C) were analyzed with repeated analysis of variance (ANOVA). Post hoc comparisons were made with Tukey’s or Sidak’s multiple comparisons test. Two-group comparison was made by t-test. All data are expressed as mean ± SEM. *p* values < 0.05 were considered statistically significant.

## Results

### PLCβ1-KO dams neglected pups after postpartum hour 18 (PPH 18)

We examined the overall behavior of PLCβ1-KO dams to decide whether and how the lack of PLCβ1 affects maternal behavior, by video-recording them continuously from the late pregnancy to a few days after parturition. PLCβ1-KO dams took as good a care of their pups (grouping, hovering/nursing; Figure 1(A) left) as wild-type (WT) dams did, but they began neglecting pups at around 18 h postpartum (PPH 18) (Figure 1(A) right) so that all pups died within 2∼3 days after birth. Plotting the relative time spent on total maternal behavior (nesting, grooming, licking, and hovering) every six hours from 24 h prepartum to 24 h postpartum showed that KO dams display normal preparation and onset of maternal behavior but did not maintain it after PPH 18 (Figure 1(B)). ANOVA [(genotype – WT, KO) X (time – nine time points from parturition)] for total maternal behavior found main effects of genotype [F(1,78) = 57.25, *p* < 0.0001] and time [F(8,78) = 24.80, *p* < 0.0001], and a significant genotype X time interaction [F(8,78) = 11.45, *p* < 0.0001]. Post hoc comparisons found a significant difference between WT and KO at PPH 18 and PPH 24 (*p*s < 0.0001). Analysis only for pup-directed behavior (licking and hovering) for 24 h postpartum showed a temporal pattern similar to Figure 1(B), revealing an abrupt decrease at PPH 18 in KO dams, which is an actual maternal neglect (Figure 1(C)). ANOVA [(genotype – WT, KO) X (time – five PPHs)] for pup-directed behavior found main effects of genotype [F(1,49) = 77.79, *p* < 0.0001] and time [F(4,49) = 10.85, *p* < 0.0001], and a significant genotype X time interaction [F(4,49) = 7.688, *p* < 0.0001]. Post hoc comparisons found a significant difference between WT and KO at PPH 18 and 24 (*p*s < 0.0001). In PLCβ1-KO dams, there were significant differences between the groups within PPH 12 and those after PPH 18 (*p*s < 0.05). Even though PLCβ1-KO mice generally do not build nests (Koh et al. 2008), the dams appeared to display nesting behavior during peripartum periods. To verify this change caused by pregnancy and maternal onset, nest construction test was performed for the four (WT, KO) X (dam, virgin) groups with commercial cotton nesting material acutely provided in the cage. Within an hour after being placed in a cage with the cotton pieces, each of all PLCβ1-KO dams tested did build a nest during the period between 24 h prepartum and PPH 12 (samples at about −24 h or PPH 6-12), but not after PPH 18 (samples at around PPH 24) (Figure 1(D)).

### PLCβ1-KO dams could not finish pup retrieval task after PPH 18

Results shown in Figure 1 suggest that PLCβ1 signaling is implicated for sustained maternal behavior after PPH 18. Casual inspection of maternity cage behaviors suggests that PLCβ1^+/-^ (heterozygous, HT) dams do not have any particular deficit in maternal caregiving. To examine whether there is a dose effect of PLCβ1 gene on maternal behavior expression after PPH 18, pup-directed maternal care was quantified in WT, HT, and KO dams with a pup retrieval test (PRT) method. In PRT, a subject is left alone with nest material first to build a new nest, and then three of its pups are placed on three corners of the cage for the subject to finish retrieving all three pups to the nest within 10 min (Figure 2(A)). After PPH 18 (samples at around PPH 24), KO dams did not even complete retrieving the first pup within the given time. HT dams completed retrieving the 1st and 2nd pup as fast as WT (*p* = 0.7711 and 0.5586, respectively), but retrieved the 3rd pup significantly slower than WT (*p* < 0.05) (t-test, Figure 2(B)).

During the period within PPH 12 (samples at PPH 6-12), KO dams retrieved each of three pups as fast as WT did during the period within PPH 12 and after PPH 18 (Figure 2(C)), which is consistent with the time course of the effect of PLCβ1 knockout on total- and pup-directed maternal behavior. ANOVA [(genotype – WT, KO) X (time – PPH12, PPH18)] for latency found main effects of genotype [1st pup, F(1,29) = 17.65, *p* < 0.001; 2nd, F(1,29) = 15.42, *p* < 0.001; 3rd, F(1,29) = 9.080, *p* < 0.05] and time [1st pup, F(1,29) = 9.970, *p* < 0.05; 2nd, F(1,29) = 10.49, *p* < 0.05; 3rd, F(1,29) = 2.763, *p* = 0.1072], and a significant genotype X time interaction [1st pup, F(1,29) = 21.91, *p* < 0.0001; 2nd, F(1,29) = 21.57, *p* < 0.0001; 3rd, F(1,29) = 8.847, *p* < 0.05] (latency values of PPH18 KO group arbitrarily taken as 600 s). Post hoc comparisons found no significant difference among PPH12 WT, PPH18 WT, and PPH12 KO groups for retrievals of all three pups (*p*s > 0.6475).

### Differential activation of brain areas in postpartum wild-type and PLCβ1-KO dams

The time course of the maternal behavioral effect of PLCβ1 knockout shown above divides the postpartum period into ‘within PPH 12’ and ‘after PPH 18’. This putative division may have implications for a role of PLCβ1 signaling for a phase transition in the maternal neurocircuitry. To find the neural basis for the abnormal maternal behavior in PLCβ1-KO dams, we first compared the neural activation patterns in the brains of KO and WT dams during these two postpartum phases, by measuring FosB expression in the medial prefrontal cortex (mPFC), nucleus accumbens (NAcc), and medial preoptic area of hypothalamus (mPOA). The immediate early gene product FosB is known to act as a major signal in parental behavior (Kuroda et al. 2007; Kuroda et al. 2008) and has been used as a neural activity marker in reward, addiction and stress systems (Hope et al. 1992; Perrotti et al. 2004; Harris et al. 2007; Gajewski et al. 2016).

**Figure 3.**
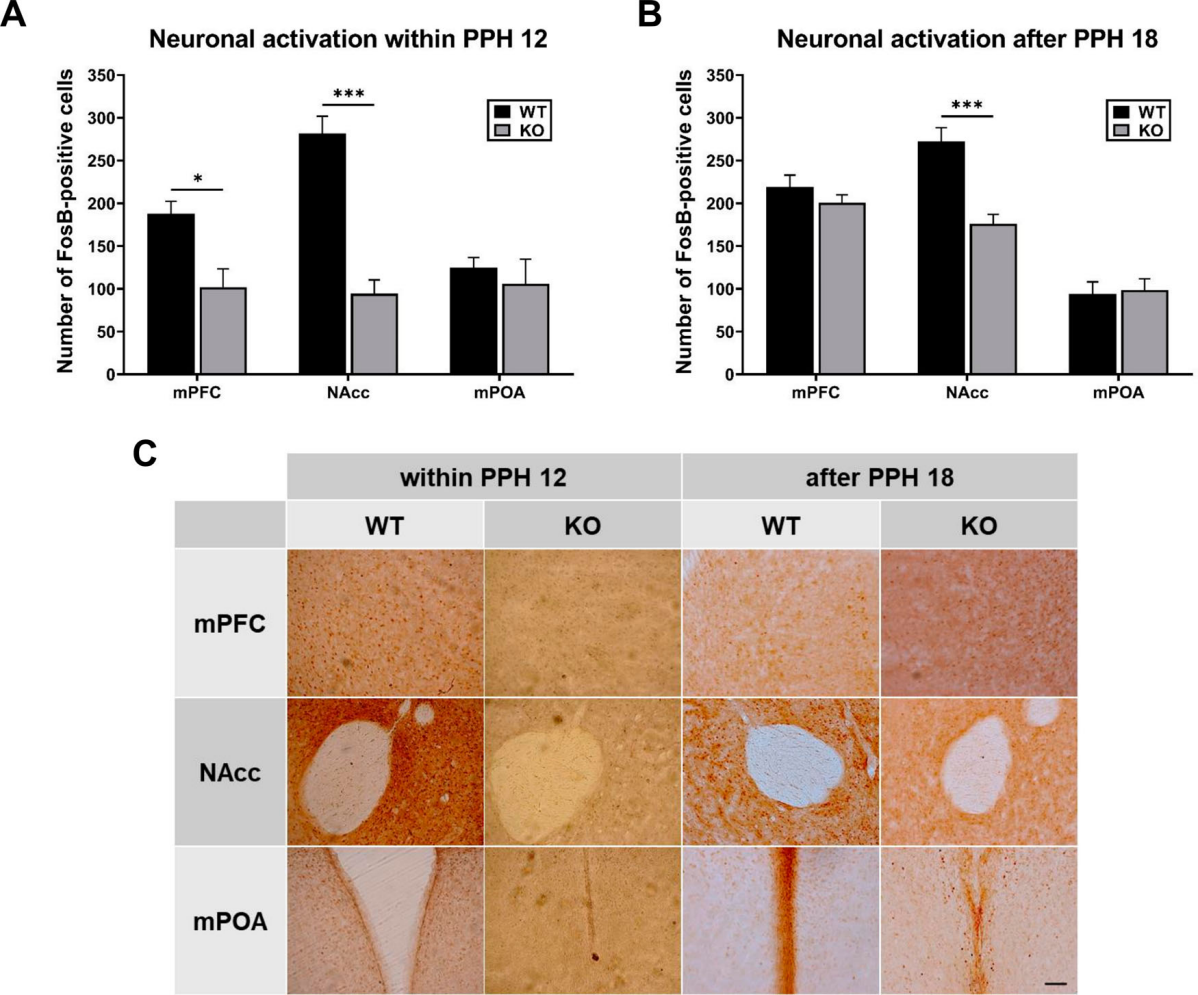
Differential activation of brain areas in wild-type (WT) and PLCβ1-KO dams within PPH 12 and after PPH 18. (A, B) Number of FosB-positive cells (per section) in the mPFC, NAcc, and mPOA of WT (black) and KO (gray) dams within PPH 12 and after PPH 18. (C) Representative images of FosB immunohistochemistry for the twelve groups from (A) and (B). Calibration bar is 100 µm. All values are Mean ± SEM. *p < 0.01; ***p < 0.0001.

During the period within PPH 12 (samples at PPH 8-12), FosB expression was significantly lower in the mPFC (*p* < 0.01) and NAcc (*p* < 0.0001) of KO dams compared with those of WT dams, and not significantly different in the mPOA (*p* = 0.4766) (t-test, Figure 3(A)). During the period after PPH 18 (samples at around PPH 24), FosB expression in the NAcc of KO dams was also significantly lower (*p* < 0.0001), and there was no significant difference in the mPFC (*p* = 0.2632) and mPOA (*p* = 0.8144) (t-test, Figure 3(B)).

PLCβ1 knockout did not affect mPOA activity both during the period within PPH 12 and after PPH 18. Given that the mPOA acts for onset of maternal behavior in response to the peripartum hormones and parturition (Numan 2012), and the normal onset observed in KO dams (Figure 1 and Figure 2(C)), these results suggest that PLCβ1 signaling is not always required for maternal initiation driven by mPOA activity. In contrast, NAcc activity in KO dams was significantly lower during both periods. The NAcc is involved in maternal motivation via mPOA output to the mesolimbic DA system (Lee et al. 2000; Numan and Stolzenberg 2009) which reinforces the primed maternal brain with pup experience postpartum. Besides, studies using postpartum rat have shown that activity of NAcc for the first 24 hs after parturition is necessary for consolidation of maternal responsiveness (Lee et al. 1999; Li and Fleming 2003). These results, together with the maternal neglect after PPH 18 observed in KO dams, suggest that PLCβ1 signaling, probably in the NAcc, is required for advancing to the putative maintenance phase. The mPFC activity in KO dams was significantly lower within PPH 12 and belatedly increased to WT level after PPH 18. Findings from studies using rats propose that the mPFC functions as an executive control system for regulation of maternal behavior (Li 2022). Therefore, it will be worthwhile to investigate the possible role of peripartum mPFC activity for transition to maternal maintenance.

## Discussion

### PLCβ1 signalling in maternal onset

Maternal care behavior is triggered by neonate stimuli immediately at parturition. This rapid onset is driven via activation of the mPOA that is primed by dynamic hormonal events of pregnancy, pregnancy termination and parturition: progesterone withdrawal, estrogen rise, prolactin (PRL) surge, and pulsatory oxytocin (OXT) (Numan and Stolzenberg 2009). mPOA contains populations of galanin neurons that suppress avoidance/aggression and promote attraction toward pups, and also have receptors for those key hormones (Wu et al. 2014; Kohl et al. 2018). The onset process is composed of many partially interacting, parallel and redundant systems such that there is no single component both necessary and sufficient to elicit immediate maternal behavior without others (Kristal 2009).

A Gαq/11-PLCβ1-coupled signal, OXT is known to be an essential regulator of maternal behavior. Both OXT-deficient (OXT-KO) and OXT receptor-deficient (OXTR-KO) mice show defects in lactation and nurturing, so that all pups die of starvation shortly after birth. However, OXT-KO mice display overall normal maternal behaviour (Nishimori et al. 1996). Both OXTR-KO and forebrain conditional OXTR-KO (FB-OXTR-KO) dams also show largely normal maternal behaviors during the onset (Takayanagi et al. 2005; Macbeth et al. 2010) but with a little more percentage of scattered pups (30%) than WT (10%) observed in OXTR-KO dams (Takayanagi et al. 2005; Macbeth et al. 2010). Although still more detailed behavioral analysis need to be done for these mutant dams (e.g. pup retrieval test during the onset phase), these results suggest that OXT signalling is involved but not completely required in maternal onset. These reports about OXT, as a PLCβ1-coupled neurohormone, are consistent with our results demonstrating normal onset in PLCβ1-KO dams. On the other hand, heterozygous PRL receptor (PRLR) KO mice cannot initiate maternal care behavior, suggesting the essential role of PRLR signal in maternal onset (Lucas et al. 1998).

### PLCβ1 signalling in maternal consolidation

Once maternal behavior becomes ‘established’ during the early postpartum hours of pup experience after the onset, its maintenance is no longer under the hormonal control. Enduring bond/attraction to pups appears to form during this consolidation of maternal responsiveness via mPOA interaction with the mesolimbic DA system (VTA to NAcc) (Numan and Stolzenberg 2009), which may cause neural/synaptic plasticity in brain region(s) that promotes goal-directed motivated responses, such as ventral pallidum (VP) (Smith et al. 2009). The current model suggests that DA input to the NAcc dampens the NAcc inhibitory output to the VP, to open the ‘pup stimuli-amygdala-VP-maternal responses’ pathway (Numan and Young 2016).

Our interpretation of the present results may lead to a hypothesis that PLCβ1 signalling is required for the putative consolidation mechanism happening in the active NAcc shell for the 24 hs postpartum. Examining behavioral effects of conditional ablation of PLCβ1 in the NAcc shell before parturition (i.e. normal onset but failed maintenance) or after 24 h postpartum (i.e. normal maintenance) may help confirm the possible role of NAcc PLCβ1 signalling for maternal consolidation. PLCβ1-KO mice display normal appetitive Pavlovian conditioning, which requires the reward process (Kim and Koh 2016, p. 2021), but no other reward behavior test has been done in PLCβ1-KO mice. Therefore, still to be decided is whether the present result was due to a general reward deficit rather than a maternal-specific one in PLCβ1-KO mice.

OXTR is densely distributed in the NAcc shell of rats (Veinante and Freund-Mercier 1997; Moaddab et al. 2015), and injection of an OXTR antagonist into the NAcc shell was shown to disrupt maternal consolidation in rats (D'Cunha et al. 2011). Pair bond formation and maintenance in prairie voles require concurrent activation of OXT and DA systems in the NAcc (Liu and Wang 2003; Loth and Donaldson 2021; Inoue et al. 2022), and OXTR is often colocalized with DA receptor D1 in the NAcc of California mice (Luo et al. 2022). These studies on pair bond of the monogamous rodents implicate the possible involvement of NAcc OXT-DA interaction in maternal consolidation in mice and rats. Maternal behaviour experiments performed on OXTR-KO dams using foster pups (i.e. after their own pups died of starvation) showed significantly slower retrieval, longer latency to crouching, and shorter crouching duration: OXTR knockout lowered the general quality of care behaviour but did not cause such downright neglect as observed in PLCβ1-KO mice (i.e. they finished retrieving all three pups) (Takayanagi et al. 2005). Therefore, still some other PLCβ1-coupled systems seem to be operating for maternal consolidation.

Another PLCβ1-coupled GPCR, vasopressin receptor type 1a (V1a) is distributed along the borderline between NAcc shell and core (Kremarik et al. 1993). V1a antagonist is also known to disrupt maternal consolidation (Nephew and Bridges 2008). On muscarinic receptor (mAChR), yet another PLCβ1-coupled receptor, no study has been known yet in relevance to any parental behavior at all, except one that reported the disruption of paternal pup retrieval by muscarinic antagonist scopolamine (Fujimoto et al. 2013). However, there are reports on muscarinic involvement in cocaine addiction (Williams and Adinoff 2008), cue-induced invigoration of reward seeking (Collins et al. 2016), and local cholinergic interneurons acting through mAChR on DA neurons in the NAcc involved in reward and motivation (Cachope et al. 2012). Given the profound effect of PLCβ1 knockout on maternal behavior, the possible involvement of NAcc muscarinic system in maternal consolidation may also need to be explored in rats and mice.
